# Physiological adaptive traits are a potential allele reservoir for maize genetic progress under challenging conditions

**DOI:** 10.1038/s41467-022-30872-w

**Published:** 2022-06-09

**Authors:** Claude Welcker, Nadir Abusamra Spencer, Olivier Turc, Italo Granato, Romain Chapuis, Delphine Madur, Katia Beauchene, Brigitte Gouesnard, Xavier Draye, Carine Palaffre, Josiane Lorgeou, Stephane Melkior, Colin Guillaume, Thomas Presterl, Alain Murigneux, Randall J. Wisser, Emilie J. Millet, Fred van Eeuwijk, Alain Charcosset, François Tardieu

**Affiliations:** 1 LEPSE, Univ Montpellier, INRAE, Institut Agro, Montpellier, France; 2 DIASCOPE, Université de Montpellier, INRAE, Institut Agro, Montpellier, France; 3 GQE-Le Moulon, INRA, Université Paris-Sud, CNRS, AgroParisTech, Université Paris-Saclay, Gif-sur-Yvette, France; 4 ARVALIS, Institut du Vegetal, Ouzouer le Marché, France; 5 AGAP institut Univ. Montpellier, INRAE, CIRAD, Institut Agro, Montpellier, France; 6 Catholic Univ. Louvain, Earth & Life Institute, Louvain la Neuve, Belgium; 7 INRAE, Univ Bordeaux, Saint Martin de Hinx, France; 8 ARVALIS, Institut du Vegetal, Boigneville, France; 9 RAGT, Port de Lanne, France; 10 MAS seeds, Haut-Mauco, France; 11 KWS SAAT SE & Co. KGaA, Einbeck, Germany; 12 Limagrain Europe, Chappes, France; 13 Biometris, WUR, Wageningen, The Netherlands

**Keywords:** Agricultural genetics, Natural variation in plants, Plant breeding

## Abstract

Combined phenomic and genomic approaches are required to evaluate the margin of progress of breeding strategies. Here, we analyze 65 years of genetic progress in maize yield, which was similar (101 kg ha^−1^ year^−1^) across most frequent environmental scenarios in the European growing area. Yield gains were linked to physiologically simple traits (plant phenology and architecture) which indirectly affected reproductive development and light interception in all studied environments, marked by significant genomic signatures of selection. Conversely, studied physiological processes involved in stress adaptation remained phenotypically unchanged (e.g. stomatal conductance and growth sensitivity to drought) and showed no signatures of selection. By selecting for yield, breeders indirectly selected traits with stable effects on yield, but not physiological traits whose effects on yield can be positive or negative depending on environmental conditions. Because yield stability under climate change is desirable, novel breeding strategies may be needed for exploiting alleles governing physiological adaptive traits.

## Abstract (web summary)

Phenomic and genomic approaches are required to evaluate the progress of breeding strategies. Here, the authors analyse 65 years of genetic progress in maize, showing that breeders have selected traits with stable effects on yield whereas not for adaptive traits key for climate change adaptation.

## Introduction

Yield progress under challenging environmental conditions is necessary to face the negative impacts of climate change and the limitations on irrigation and fertilization, in a context of high demand for agricultural products^1,2^. Breeding and the improvement of agronomic practices have increased yields of most species^1,3^. In maize, a major source of yield progress was the fine-tuning of plant cycle duration to the local environmental conditions, based on the well-documented genetic variability for flowering time^4,5^. However, the genetic ability of varieties to produce high yield has also rapidly increased in maize, for a given cycle duration and a given set of management practices, representing 50–75% of total yield gain^6–14^. For each class of crop cycle duration, this gain was essentially obtained by breeders via selection for yield and, more recently, genomic selection based on yield^15,16^.

A crucial question is whether this rate of genetic progress can be sustained in the next decades. Evaluating the margin of the progress of breeding strategies requires a comprehensive analysis that links, in the same genetic material, yield progress to underlying traits and corresponding genomic regions. This would allow the evaluation of which traits can still be improved, in relation to likely physical limits, and the allele fixation achieved for these traits by breeding programs. Elements for this exist in the literature, either for identifying the genomic changes associated with selection^17,18^, to evaluate the yield progress over time of released varieties in multi-site field experiments^6–13^, or to identify traits that appreciably changed with year of release^7,19,20^. To our knowledge, the whole path of examining causal links between the changes in alleles, traits, and yield in a range of environmental scenarios has never been analyzed in the same genetic material. This path is not straightforward because a vast majority of yield quantitative trait loci (QTLs), which necessarily act via underlying traits, have positive, negative, or neutral effects depending on environmental conditions^21,22^. This justified the fact that we considered trait effects on yield in a range of environments, but also that we characterized their changes across generations of selection.

Different categories of traits are potentially involved in genetic progress. Adaptive physiological traits (e.g., stomatal control or leaf growth) vary several-fold over minutes to hours as a response to environmental conditions for a given genotype^23^, so it is challenging to consider them in a breeding program (in addition to the intrinsic difficulty to phenotype them at high throughput^24^). We recently proposed that evolution has coordinated the numerous and complex adaptive mechanisms into strategies ranging from ‘growth-oriented’ to ‘conservative’, which favor yield in most favorable vs most adverse environmental conditions^23,25^. Constitutive traits (e.g., duration of phenological phases expressed in thermal time or plant architectural traits, including harvest index) have long-term variations with the environment, so they are selectable in a breeding program^15,23^. Their effect on yield is less dependent on environmental scenarios than adaptive traits, with the notable exception of crop cycle duration (expressed in thermal time), for which shortest durations allow escaping stress in driest or hottest scenarios, whereas longest durations compensate the effects of increased mean temperatures in the absence of water deficit^5^.

In this study, we evaluated the contributions of a range of traits and alleles to the genetic progress of yield, in particular to what extent yield-based selection indirectly affected adaptive, environment-dependent traits and constitutive traits having more stable effects. In a maize panel of 66 European varieties released from 1950 to 2016, we (i) measured physiological traits with novel phenomic methods, in particular stomatal conductance at the plant level, growth responses to environmental conditions, plant architecture, and reproductive development (see Supplementary data 11 for precise definitions of variables), together with plant phenology in ten high-precision experiments in three phenotyping platforms^24,26,27^ and three equipped fields (Supplementary Table 2), (ii) measured yield and its components in 30 field experiments across Europe (Supplementary Table 2), (iii) analyzed trait contributions to yield improvement via linear models and Bayesian networks, and (iv) investigated if genomic regions associated with these traits showed signatures of selection. Such signatures were sought in the same panel of genetic progress as above, whereas genomic regions associated with studied traits were those previously identified in a diversity panel with a similar genetic background compared to that of the genetic progress panel studied here (Supplementary Fig. 1). Finally, we examined a hypothesis that adaptive traits did not respond to selection due to their conditional effects on yield.

## Results

### The studied environments and hybrids were typical of the growing area of European maize

**Fig. 1:**
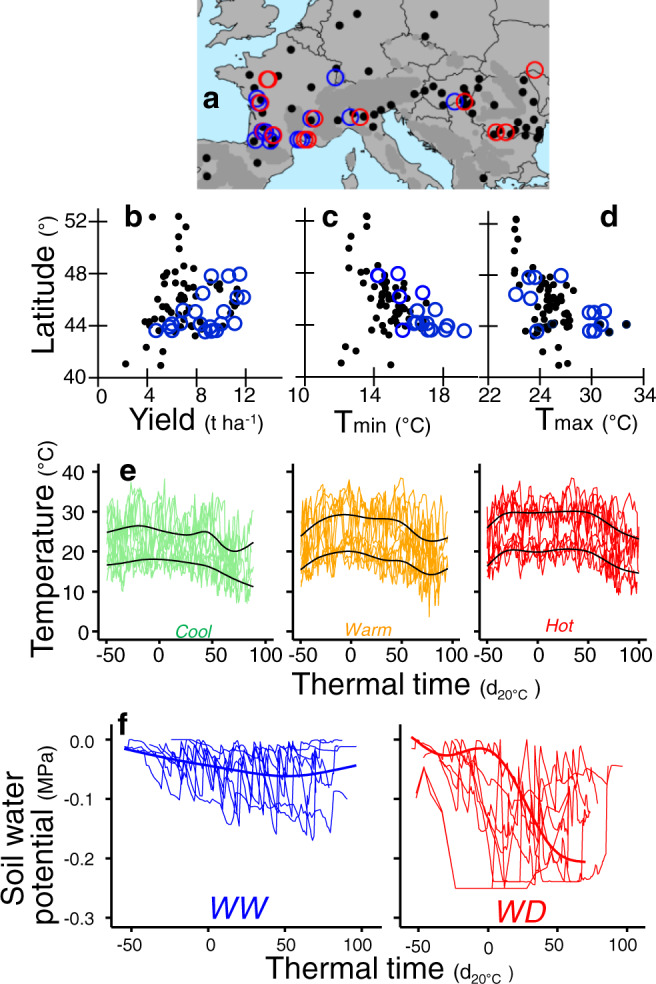
The field experiments covered the latitudes with maximum area of maize in Europe, and represented a large part of the variability of yield and environmental conditions. **a** Map of field experiments, blue and red circles for well-watered and water deficit experiments, and of 59 sites distributed over Europe for representation of the maize growing area. **b**–**d** Yield, minimum and maximum temperatures in experiments as a function of latitude, compared with means for 59 sites over 35 years. **e** Three temperature scenarios captured a large part of the variability of temperature in the field experiments. Time courses of air temperature in the scenarios cool, warm, and hot. Each line represents one field, black lines, mean time course. **f** Time courses of soil water potential for fields classified as belonging to well-watered or water deficit scenarios. Each line represents one field. In **e** and **f**, time is centred on flowering time, in equivalent days at 20 °C (d_20 °C_). Experiments i, j, l and m, see supplementary Table 2. Source data in Supplementary data 1.

The genetic progress was analyzed by comparing traits and performances of the 66 most commercially successful European hybrids released from 1950 to 2015 (Supplementary Table 1). Because each maize variety grows in a limited range of latitude, we chose the range 43–48°N which covers a large proportion of the maize growing area in Europe (Fig. 1a)^5^, and corresponds to mid-early (FAO 280) to mid-late (FAO 490) hybrids.

This genetic progress panel captured the elite breeding pools of European and American germplasm, as shown by projecting the genotypes onto a principal component biplot of 96 commercial varieties with expired Patent Variety Protection (ex-PVPs; Supplementary Fig. 1). Notably, an appreciable shift in SNP frequency was observed with year of release in the genetic progress panel, with an increasing proportion of Iodent material (Supplementary Figs. 1 and 2, Supplementary Table 1). In order to leverage additional data, we also compared the genetic progress panel to a diversity panel of 250 hybrids^21,28,29^ which covers the whole domain of the PCA for ex-PVPs, and was extensively phenotyped^21,30–33^. Importantly, this diversity panel served as a training population for genomic prediction of yield in a European breeding population^34^, lending credence to comparative analysis of QTLs identified in this panel with signatures of selection in the genetic progress panel (described later).

**Fig. 2:**
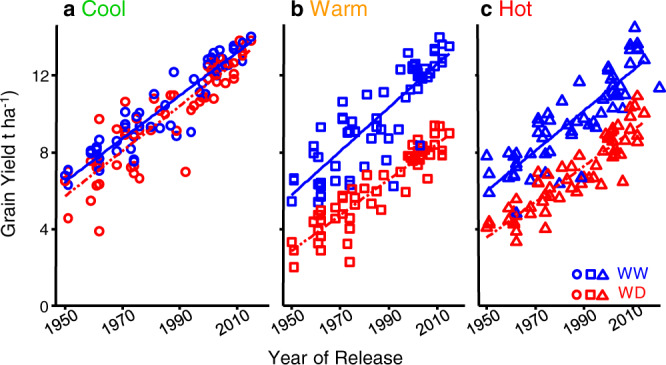
The genetic gain of yield was similar in all studied environmental scenarios (26 field experiments). Change in grain yield for maize hybrids released from 1950 to 2016 in six environmental scenarios presented in **a**–**c**. Results of experiments i, j, l, m (not k) as in supplementary Table 2. Regression lines are drawn when slopes are significant (*p* < 0.01). WW well-watered, WD water deficit. Source data and *p*-values in Supplementary data 2.

Mean yields in the 30 studied experiments covered the entire yield range simulated over 35 years in 59 sites in Europe^5^ (3–12 t h^−1^, latitudes 41–53°N, Fig. 1b). The measured environmental conditions recorded in studied fields also captured the wide range of European-scale conditions (Fig. 1e, f). Field experiments were clustered in six environmental scenarios defined by combinations of favorable to highly unfavorable air temperature, evaporative demand, and soil water status (Fig. 1e), which markedly affected yield (Fig. 2). Overall, minimum and maximum temperatures during the flowering time covered the major range observed for the European maize growing area over the last 35 years (e.g., maximum temperatures 24–32 °C in our experiments vs 24–29 °C for the 59 × 35 studied records in European sites Fig. 1c, d). They tended to be higher than those measured at the same latitudes in the last 35 years, potentially a consequence of climate change. Hence, the genetic progress was studied here in a range of climatic conditions that essentially covers both the current and past climatic conditions in the European maize growing area, avoiding the bias recently suggested when old hybrids are compared with recently released hybrids in today’s environments only^35^.

### The rate of genetic progress was high and similar in fields with high/low temperatures or water status, and at two plant densities

**Fig. 3:**
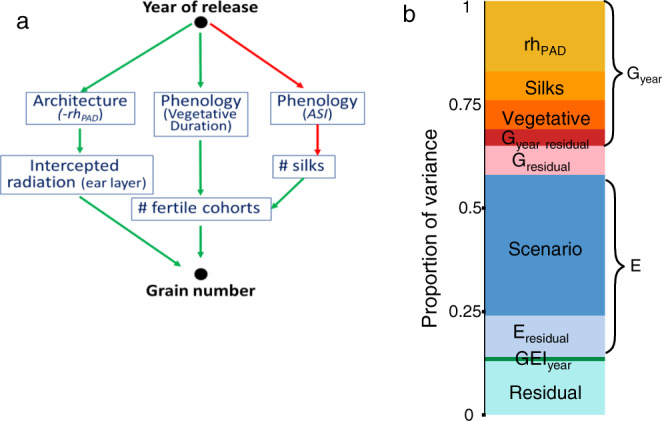
The year of release affected grain number via phenology and plant architecture. **a** Bayesian network analysis of conditional dependencies between year of release, grain number, and phenotypic traits. Presented arrows are consensus in all environmental scenarios, green and red: positive (resp negative) relationships. rh_PAD_, distance, from top of the canopy, of the layer with 50% of total leaf area, in % of canopy height. Intercepted radiation (ear layer), amount of light intercepted by the canopy layer hosting ears, normalized by incident light. # fertile cohorts, number of ovary cohorts that became grains. ASI anthesis-silking interval. **b** Proportion of the variance of grain number accounted for by traits contributing to the genotypic main effect, by environmental main effect and by the interaction between genotype and environment. The analysis first distinguished the proportion of variance explained that depended on year of release (orange rectangles) or non-accounted by it (pink). The proportion of variance explained by year of release was then split into traits: duration of the vegetative period (‘vegetative’), number of emerged silks (‘silks’) and architecture (‘rh_PAD_‘). G_year residual_ represents the proportion of the variance accounted for by year of release but none of the above traits. E—proportion of the variance accounted for by environmental scenarios and the residual environmental effect. In GEI, the dark green rectangle represents the interaction between year of release and environmental scenario, the light green rectangle represents the residual G × E and error. Source data in Supplementary data 3.

The annual genetic gain in grain yield (i.e., the slope of the regression between yield and year of release) was, on average, 101 kg ha^−1^ year^−1^ (*n* = 60, *p*-values < 10^−5^) representing 75% of on-farm yield increase in Europe for the same period^3^. It was as fast in favorable scenarios as in unfavorable ones with water deficit or high temperatures (Fig. 2). Indeed, the interaction between the year of release and the environmental scenario accounted for only 0.9% of yield variance (GEI_year_, Fig. 3b), consistent with earlier studies^7,16^. The genetic gain was also similar at two plant densities representative of farmers’ practices in 1960 vs 2010^36^ (7 and 9 plants m^−2^, Supplementary Fig. 3), corresponding to different competition for light and soil water. This result was unexpected because breeding for high plant density was proposed as a key mechanism for maize genetic progress^7,36,37^.

With grain number per unit area and individual grain weight as key components of maize yield, the genetic progress in yield was mainly driven in all scenarios by an increased grain number, fixed at the end of flowering^38^ (Supplementary Fig. 4g–i), consistent with earlier studies^39,40^. Year of release (G_year_ in Fig. 3b) captured 74% of the genetic effect on grain number in the considered panel. Environmental scenarios (Scenario in Fig. 3b) captured 76% of the environmental effect (Fig. 3b, see also boxplots in Supplementary Fig. 5). Individual grain weight, dependent on the grain-filling phase, progressed by 14% with little difference between scenarios and was weakly related to yield (Supplementary Fig. 4).

### A change in phenology resulted in an improvement in reproductive development

**Fig. 4:**
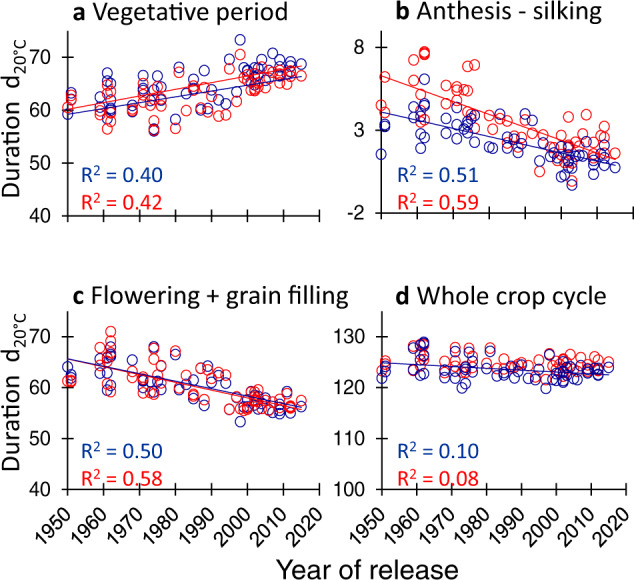
Change with year of release of the phenological phase durations. **a** Vegetative period, from emergence to anthesis, **b** anthesis-silking, from anthesis to silk extrusion from the ear, **c** flowering + grain filling, from anthesis to maturity, **d** whole crop cycle, from emergence to maturity. Field experiments i, j, l and m. Blue symbols, experiments with soil water potential higher than −0.1 MPa throughout the plant cycle; red symbols, experiments with water deficit. One dot per genotype × soil water scenario (BLUEs). Regression lines are drawn when slopes are significant (*p* < 0.01). For better intuition, panels **a**, **c**, **d** are presented with the same scale. Source data and *p*-values in Supplementary data 4.

**Fig. 5:**
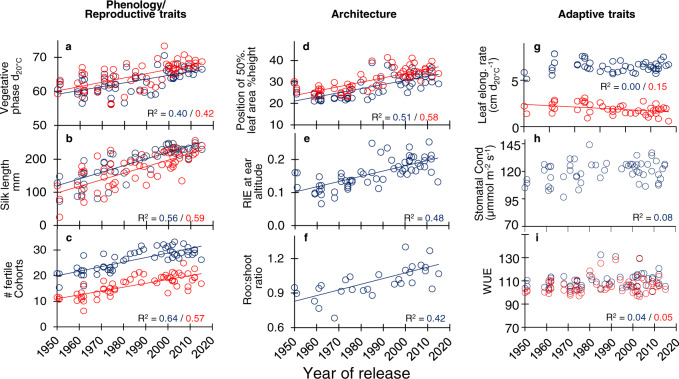
Phenological, reproductive, and architectural traits changed with year of release similarly in all environmental conditions; adaptive traits did not. **a** Duration of the vegetative period. **b** Mean silk length at anthesis (from insertion point to silk apex). **c** Number of fertile grain cohorts per plant. **d** Distance, from top of the canopy, of the layer with 50% of total leaf area, in % of canopy height. **e** Amount of light intercepted by the canopy layer where ears are located, normalized by incident light (unitless). **f** Root:shoot ratio (unitless). **g** Leaf elongation rate (LER). **h** Whole-plant stomatal conductance. **i** Water-use efficiency. Blue dots, well-watered plants (soil water potential > −0.1 MPa) and red dots, water deficit (soil water potential < −0.1 MPa). Each dot is the BLUE value corresponding to one hybrid. Origin of data: **a** Exp i, j, l, m (Supplementary Table 2); **b** platform Exp a. **c** Exp i, j, l, m. **d**, **e** platform Exp a. **f** platform Exp h; **g** platform experiments d to f. **h** platform Exp g. **i** platform Exp a. See Supplementary Figs. 4 and 5 for other experiments. Regression lines are drawn when slopes are significant (*p* < 0.01). Source data and *p*-values in Supplementary data 5.

**Fig. 6:**
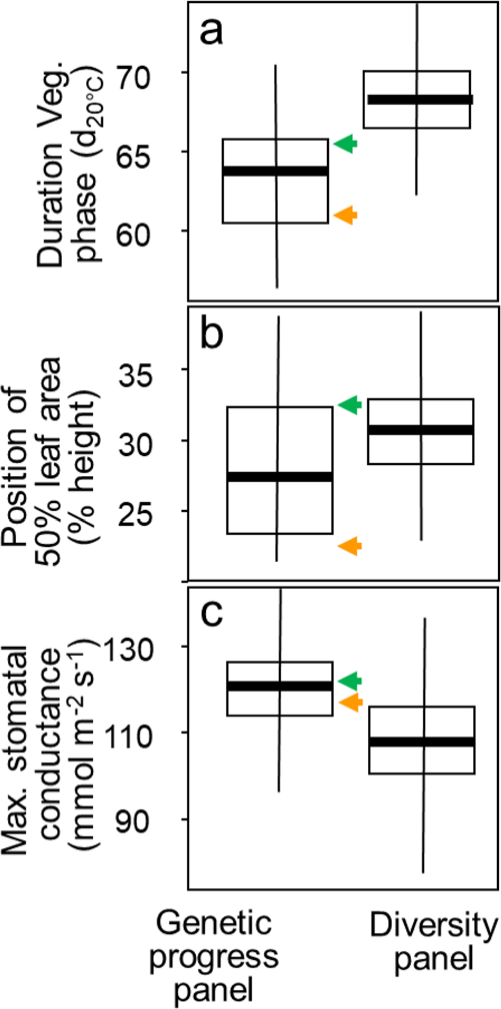
The genetic progress (green and orange arrows) accounted for the largest part of the genetic variabilities of vegetative phase duration and of architecture, and for a small part of the genetic variability of stomatal conductance. Left boxes, genetic progress panel (presented here); right boxes, diversity panel with similar background. Green and orange arrows, median of the phenotypic value of the 22 most recent and 22 most ancient hybrids, respectively. Phenotypic values taken into account are the BLUES for each hybrid. **a** Duration of the vegetative phase; **b** Distance of the canopy layer with 50% of total leaf area from top, in % of canopy height (rh_PAD_); **c** maximum stomatal conductance. *n* = 56, 60, and 60 hybrids in panels **a**–**c**, respectively. Bold lines in boxes, median; boxes, 25 and 75 percentiles; vertical lines, 25 and 75 percentiles multiplied by 1.5 of the interquartile range (25–75). Source data in Supplementary data 6.

Breeders indirectly changed plant phenology by selecting for yield. While crop cycle duration was essentially constant with year of release (Fig. 4d, slightly decreasing duration *p*-value = 0.01 and 0.02 in WW and WD, respectively), opposing changes were observed in the duration of vegetative (longer by +10 equivalent days at 20 °C, Fig. 5a, *p*-value < 10^−5^) and grain filling (shorter, *p*-value < 10^−5^) phenological phases (Fig. 4c). The genetic variability of the duration of the vegetative phase was largely exploited by breeding, because the difference between the oldest and newest hybrids covered most of it, in both the genetic progress and the diversity panel (Fig. 6a). The increased vegetative phase duration was coupled with a reduction in the anthesis-silking interval (ASI, Fig. 4b, *p*-value < 10^−5^) which acted independently on grain number, as shown by Bayesian network analysis (Fig. 3a). Individual grain weight was maintained across years of release despite a shorter duration of the grain-filling period, so the rate of grain filling per unit time was improved by breeding. This was either because of genetic improvement of carbon translocation, or because photosynthesis during the grain-filling period was improved since grain filling is essentially sink-limited.

**Fig. 7:**
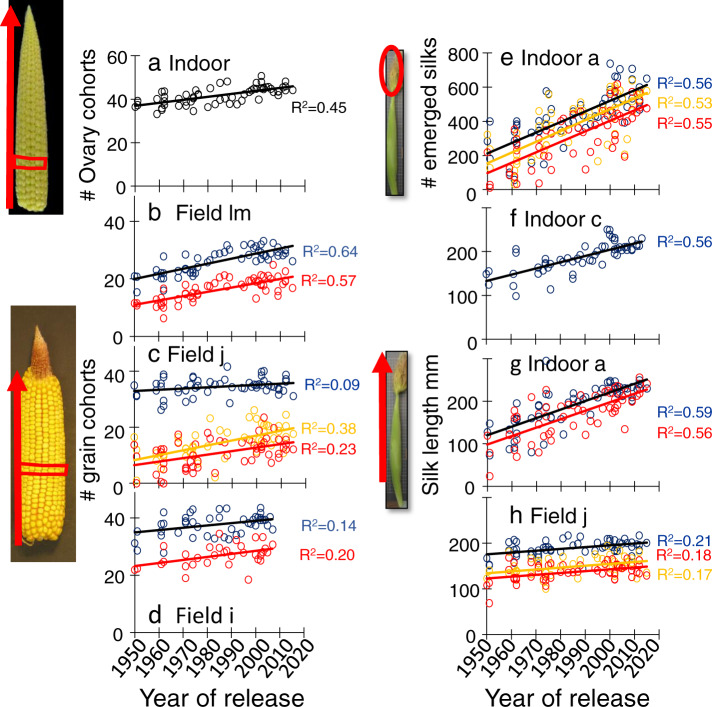
Traits related to reproductive development changed with similar rates in all studied environments. **a** Number of ovary cohorts, initiated from the base of the ear (red arrow). **b**–**d** Number of grain cohorts. **e** Number of silks extruding from the husks at anthesis. **f**–**h** Silk length at anthesis, from insertion point to their tip. In **b**, blue and red dots for the water scenarios of multi-site experiments as in Fig. 1. In **c**, blue, orange, and red dots: well-watered, medium water deficit and strong water deficit. In **d**, blue and red symbols: well-watered and water deficit. One dot per hybrid (BLUEs). In **e**–**h**, blue dots for well-watered (soil water potential higher than −0.1 MPa), red dots for water deficit, green triangle for well-watered plants with heat stress and orange dots for intermediate soil water deficit. **a**–**h** One dot per hybrid (BLUEs). Regression lines are drawn when slopes are significant (*p* < 0.01). Source data and *p*-values in Supplementary data 7.

Reproductive development was profoundly affected by these changes in phenology, as assessed in phenotyping platforms and detailed field experiments (Fig. 7). Firstly, ovary cohorts, which sequentially develop along with the ear, increased in number with year of release in all studied treatments for soil water status and temperature (Fig. 7a, Supplementary Fig. 6a, *p*-values in the figure legend). The prolonged vegetative phase observed in recent varieties allowed more ovary cohorts to be initiated^38,41^. Second, silk growth and the number of extruded silks, which determine the ASI^42^, increased with year of release in all fields, platform experiments, and treatments (Fig. 7e–h, Supplementary Fig. 6a). This contributed to increasing the proportion of ovary cohorts that become grains, via a reduced ovary abortion^38,41,43^. The network analysis in Fig. 3a suggests that the increased vegetative duration (supporting more ovaries) and the increased silk growth (reducing abortion rate) mostly contributed to the observed increase in the number of fertile grain cohorts for modern hybrids, which was observed in all field experiments (Fig. 7b-d). Interestingly, the number of grains or ovaries per cohort, which is independent of the duration of the vegetative phase, remained unchanged (Supplementary Fig. 7). This reinforces the hypothesis of a phenology-driven change in reproductive development underlying the yield progress for elite maize hybrids. Overall, changes in phenology and its consequences on reproductive development captured 40% of the variance explained by genetic progress on grain number (Fig. 3b).

### A change in plant architecture favored light interception at ear level in spite of unchanged or reduced leaf area

The second set of traits contributing to yield progress related to plant architecture (48% of variance explained by genetic progress, Fig. 3b). Virtual 3D reconstructions were built every day to replicate plants of each hybrid measured in the phenotyping platform^44^. The vertical distribution of leaf area, calculated for each plant, appreciably changed with a year of hybrid release, with an increased proportion of leaf area located at lower altitudes in the canopy (Fig. 5d, rh_PAD_ in Fig. 3a, *p*-value < 10^−5^). The difference between the oldest and newest hybrids accounted for most of the genetic variability in rh_PAD_ in the genetic progress and diversity panels (Fig. 6b). Therefore, breeding for yield improvement also indirectly affected this trait. This was due to larger leaves inserted at the lowest positions on the stem^44^, together with increasingly erect leaves also observed in our field experiment (Supplementary Fig. 9c, d) and in other studies^18,45,46^. In turn, this changed the vertical distribution of intercepted light, calculated via a model of light penetration in virtual field canopies at three plant densities, made-up of repetitions of each individual hybrid tested^44,47^. This model^44^ and the network analysis (Fig. 3a) showed that the change in rh_PAD_ increased the amount of light intercepted by the canopy layer harboring ears (Fig. 5e, Supplementary Fig. 8a), which provides most of the carbon supply to the growing ear^48^. Grain development was therefore improved, thereby avoiding grain abortion^41,49^ and, in turn, increasing grain number per unit of radiation intercepted by the whole canopy (Supplementary Fig. 10a). The root:shoot ratio, measured in an aeroponic phenotyping platform, and the radiation use efficiency calculated via a 3D model^44^ both increased in modern hybrids (Fig. 5f, Supplementary Fig 8b). This was due to changes in allometries of roots vs shoots and of the whole plant vs leaves. Indeed, shoot biomass and leaf area tended to decrease at a given date with year of release (Supplementary Figs. 8, 9, 11, 12). Notably, the leaf area at the adult stage was still highest in the most recent hybrids because it grew for a longer duration during the vegetative stage (Supplementary Fig. 9b).

### Leaf growth sensitivity, stomatal conductance, and water-use efficiency were essentially unaffected by selection in spite of large genetic variabilities and heritabilities

Studied traits related to adaptation to water deficit, evaporative demand, or high temperature showed little change with year of release (Fig. 5g-i). Stomatal conductance, calculated at the plant level for all hybrids in a phenotyping platform by model inversion^30^, presented no significant trend across the year of release (Fig. 5h, *p*-value = 0.03), consistent with an essentially stable biosynthesis of the stress hormone abscisic acid^50^. Stomatal conductance was therefore similar in the oldest and newest hybrids in spite of a large genetic variability observed in both the genetic progress and diversity panels, with a high heritability^30^ (Fig. 6c).

Leaf elongation rate, extremely sensitive to short-term environmental variations^51^, tended to slightly decrease with year of release in water deficit whereas it was stable in well-watered conditions, indicating an essentially stable drought sensitivity of leaf growth (Fig. 5g, *p*-values = 0.95 and 0.005 in WW and WD, respectively). As in the case of stomatal conductance, the large genetic variability was not exploited by breeding as shown by close leaf elongation rates in modern vs old varieties (Supplementary Fig 6b). Water-use efficiency i.e., the ratio of biomass to transpiration, largely affected by stomatal behavior, was also stable in spite of high genetic variability and heritability (Fig. 5i, *p*-values = 0.11 and 0.10). Finally, the relation of grain yield to cumulated transpiration at harvest simulated via a crop model^52^ showed a change in generational responses to transpiration that was a by-product of increased grain yield and not the consequence of better transpiration efficiency (Supplementary Fig. 13). The absence of strong or significant trends for these traits, and presumably for others that were not studied here, resulted in an essentially stable sensitivity of grain number to soil water status, with a slight tendency to increase in the multi-site field experiment (Supplementary Fig. 10b). This is despite large genetic variability and heritability for sensitivity to soil water status in both genetic progress and diversity panels, and its genomic prediction in the diversity panel^29^.

### Phenology and plant architecture showed signatures of selection whereas physiological adaptive traits did not

We tested if signatures of selection in the genome were consistent with the phenotypic trends presented above. For that, we first identified regions of the genome that showed evidence of selection in the genetic progress panel. We then compared the resulting regions with published QTLs for the traits presented above, identified in the diversity panel^21,30,32^. The rationale was to evaluate to what extent the genetic diversity available at genomic regions associated with the studied traits has been exploited by breeders.

**Fig. 8:**
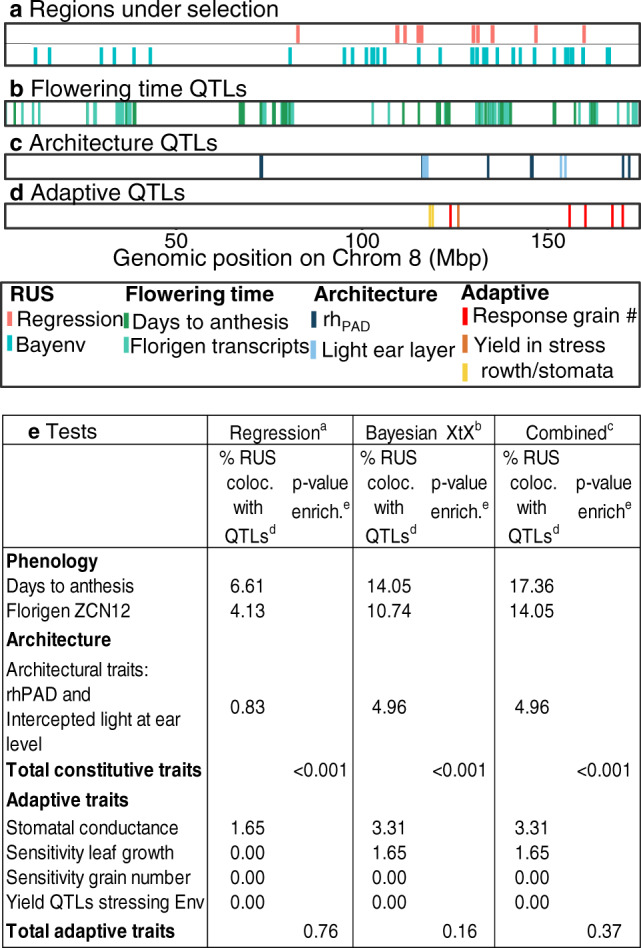
Regions under selection (RUS) colocalized with QTLs of phenology and architecture traits, not with QTLs of adaptive traits. **a** Regions under selection (RUS) in Chrom. 8 identified by (i) differentiation between the 22 most ancient and 22 most recent or (ii) regression of allelic values with year of release. **b**–**d** Known QTLs involved in three categories of traits. **e** Proportion of RUS colocating with QTLs, and Chi2 test. Other chromosomes are presented in Supplementary Fig. 11. In **e**, results of two independent methods.^a^ SNPs with −log_10_ (*p*-value > 3.5 in the regression of allelic values with year of release, whole genome. ^b^ Top 0.05% Bayenv XtX^37^ based on the differentiation of the 22 most recent and 22 most ancient hybrids. ^c^ RUS identified by combining methods. ^d^ Percentage of resulting RUS that colocalize with considered QTLs, and *p*-value of enrichment: comparison with 100,000 random genomic regions of same size at QTL regions. *N* = 60. Source data in 10.15454/KLD0GH/ genotypic dataset for the physical map and in corresponding papers for QTLs.

Regions under selection (RUS) were detected (Fig. 8) based on two methods, (i) a genome-wide scan for adaptive divergence between old and modern hybrids was performed using a Bayesian statistic^53^, and (ii) for all studied hybrids, a regression analysis of reference (B73) allele counts on the year of release was performed (Supplementary Fig. 2). Both methods accounted for genetic covariance among individuals and therefore the global trends observed in Supplementary Figs. 1-2). This allowed for the identification of genomic regions with robust differentiation across decades of selection.

We performed a meta-analysis of the RUS loci together with QTLs for phenological, architectural, or adaptive physiological traits identified in the diversity panel (Fig. 8), measured in the same set of phenotyping platforms and a similar field network used for the genetic progress panel^21,30,32,33^. The analysis included QTLs for: (i) flowering time and related traits^33^, (ii) florigens (eQTLs)^33^, (iii) stomatal conductance and difference in biomass between well-watered and water deficit treatments^30^, (iv) radiation interception efficiency (RIE) and vertical distribution of leaf area^26^, (v) sensitivity of leaf growth to soil water potential, (vi) sensitivity of grain number to soil water potential, night temperature, and light intensity^29^, also characterized by QTLs of grain yield observed only in fields with high temperature or water deficit^21^.

We then estimated the proportion of RUS overlapping with QTLs for individual traits and tested for enrichment of the observed data relative to an expected distribution from 10^5^ random iterations of genomic regions having the same number and length (tested for both physical and genetic scales) as the QTL space for each trait. Regions under selection largely overlapped with QTLs for flowering time, florigens, and architectural traits (Fig. 8, Supplementary Fig. 14), more than what would be expected by chance alone. Moreover, the reference allele frequency differences between old vs recent hybrids at locations overlapping with these QTLs were significantly greater than for random regions in the genome. Conversely, QTLs for stomatal conductance, leaf growth rate, or water-use efficiency were found to scarcely colocalize with RUS, and the QTL space for these traits showed little difference in allele frequencies between old vs recent hybrids.

### Why physiological adaptive traits may not have been selected: a fluctuation of allelic effects with environmental scenarios

**Fig. 9:**
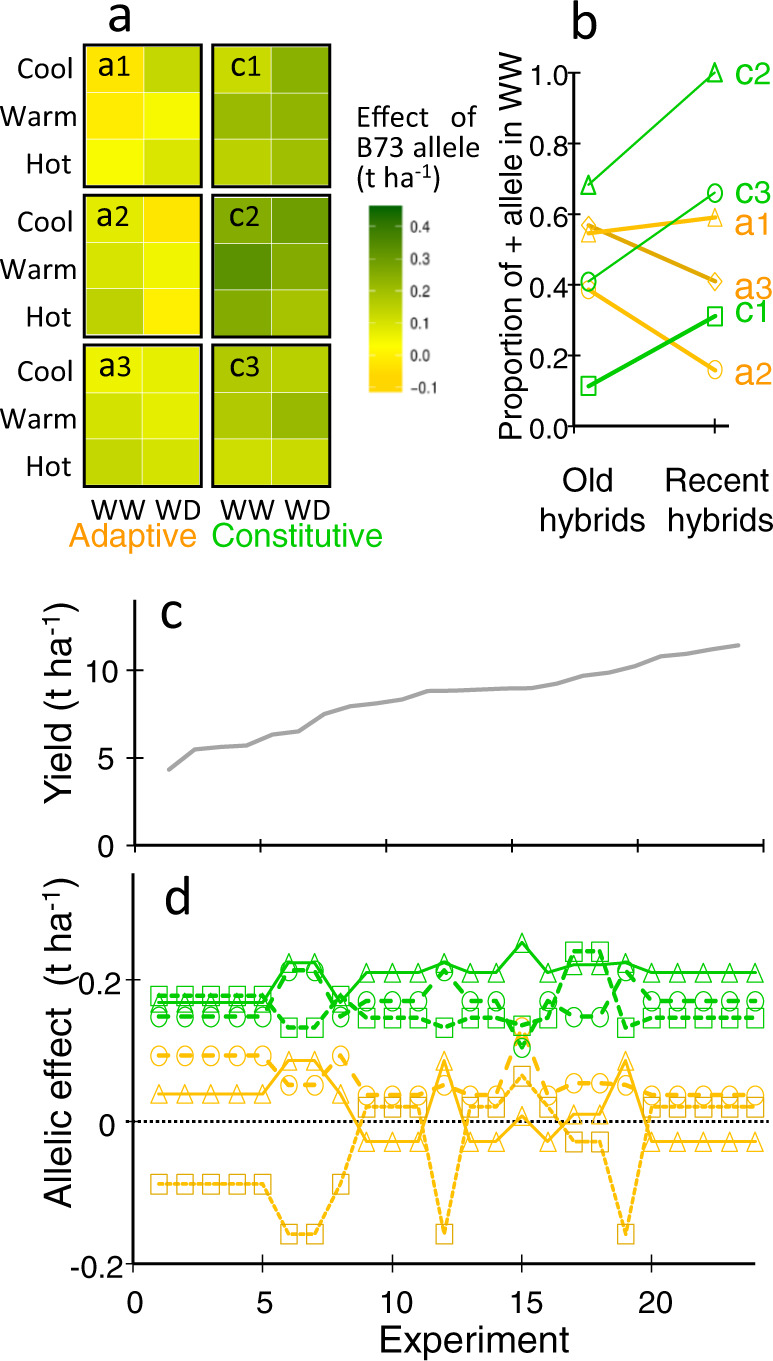
Why adaptive traits were not selected by yield-based breeding? Instable effects on yield may have caused non-selection of corresponding alleles. Effects of the six QTL are presented in **a**, averaged per environmental scenarios as in Millet et al. 2016. The proportions of positive alleles at adaptive (orange) vs constitutive (green) QTLs are presented in **b** for the 22 oldest hybrids vs the 22 most recent hybrids of the genetic progress panel. **d** Allelic effects on yield, predicted in the multi-site experiments ordered by increasing yield (**c**). QTLs with stable effects (green, constitutive traits) vs QTLs with context-dependent effects (orange, adaptive traits). Adaptive QTLs: a1, QTL on Chrom 2, 44.5 Mb affecting ABA synthesis and including an NCED gene; a2, QTL on Chrom 5, 5.4 Mb, colocalizing with maximum stomatal conductance; a3, QTL on Chrom 10, 132 Mb, whose effect increases with temperature; Constitutive QTLs: c1, QTL on Chrom 6, 84.4 Mb, colocalizing with a QTL of rh_PAD_; c2, QTL on Chrom 8, 159 Mb, colocalizing with QTLs of flowering time in field and controlled conditions, and with an eQTL of florigens ZCN8 and ZCN12; c3, QTL on Chrom 1, 2.4 Mb. Source data in Supplementary Data 8.

We examined a possible explanation why physiological adaptive traits were not selected in the breeding programs behind the genetic progress panel studied here, despite the fact that these traits can have appreciable impacts on yield under given environmental scenarios^23^. For this purpose, we selected six published yield QTLs detected in the diversity panel that either (i) correspond to adaptive traits (biosynthesis of abscisic acid or stomatal conductance) with allelic effects affected by water deficit (Fig. 9a QTLs a1 and a2) or high temperature (Fig. 9a, QTLa3); or (ii) correspond to flowering time or architectural traits with essentially stable allelic effects on yield (Fig. 9a, QTLc1 to c3). We used the allelic effects estimated in the diversity panel in each scenario (Fig. 9d) to predict the potential QTL × scenario interaction in the multi-site field experiment presented here. For that, each field experiment studied here was assigned to its respective environmental scenario so the corresponding allelic effect was assigned to each QTL.

The predicted allelic effects on the yield of the three physiological adaptive QTLs were highly variable in the multi-site field experiment, ranging from −0.16 to +0.17 t h^−1^ depending on the QTL and field (±3% of yield in unfavorable conditions in which these QTLs have largest effects). These allelic effects would also oscillate in each field with the year-to-year variability of climatic conditions^54^. These oscillations may explain why breeding programs did not affect, for these QTLs, the frequencies of the allele with a positive effect on yield under favorable conditions. Indeed, these frequencies were stable or decreased between the 22 oldest and the 22 most recent hybrids (Fig. 9b). Conversely, the predicted allelic effects on the yield of the three developmental or architectural QTLs were more stable, from 0.10 to 0.24 t ha^−1^, with a mean effect of +0.18 t ha^−1^ (2% of mean yield, Fig. 9d). Consistently, the allelic frequencies of the positive allele for yield in favorable conditions increased, for these genomic regions, between old and recent hybrids (Fig. 9b). Interestingly, breeding eliminated the unfavorable allele for yield at the most stable QTL (Fig. 9a, b, QTL c2).

These results suggest that breeding for yield may indirectly select for constitutive traits such as flowering time or rh_PAD_, corresponding to QTLs with stable effects. In contrast, the physiological adaptive traits were not selected because they correspond to QTLs with scenario-dependent allelic effects. Indeed, the coupling of a crop model with a model calculating the change in allelic composition in a breeding population suggests that the allelic frequencies at QTLs with unstable effects oscillate between years without any clear trend, which is not the case for QTLs with more stable effects^54^.

## Discussion

This study results in the somewhat paradoxical conclusion that physiological adaptive traits, whose tight control is essential for the survival and reproduction of plants under stress (e.g., the control of stomatal conductance or of growth under water deficit), have not been affected by breeding in the considered elite pools of temperate varieties. In natural contexts, avoidance of soil water depletion via limitation of transpiration in the early stages of the plant cycle allows the production of at least a few fertile grains. In agricultural contexts, yield improvement in drought-prone areas was reached via ‘conservative’ agronomical practices that limit transpiration, such as reduced duration of crop cycle or skipping one plant row out of two^5,55^. Here, we observed yield improvement under water deficit or high temperature, showing a stable yield gap with parallel progress caused by the same traits as in favorable conditions. Selection on yield indirectly improved essential traits, such as reproductive development and the conversion of light into biomass, in all studied environmental conditions. Conversely, physiological adaptive traits resulting in conservative water use were not selected, even for mostly dry areas.

A first possible interpretation might be that physiological adaptive traits bring yield advantage only in extreme conditions mostly incompatible with agriculture. However, a strong genetic variability and high heritabilities were observed for stomatal conductance and growth sensitivity in the diversity panel^30^, in which all lines derive from landraces used by farmers in the past^28^. Genetic differences and high heritabilities were also observed for stomatal conductance, water-use efficiency, and ABA synthesis in several breeding populations^56,57^. Hence, we propose that the genetic variability within the temperate maize breeding pool was not exploited by breeding strategies in the last 60 years.

A second hypothesis is that our conclusion is specific to the breeding process in Europe, which would not favor the selection of traits that confer adaptation to adverse conditions. However, the retrospective analysis of maize yield over Europe (Fig. 1b) shows that yields lower than 6 t ha^−1^ on average over 30 years were observed at 32% of the sites (15% lower than 5 t ha^−1^). Breeding companies involved in this study state that their own multi-site breeding experiments include such low-yielding fields (Stephane Melkior, Colin Guillaume, Thomas Presterl, and Alain Murigneux, personal communication). Hence, the selection pressure that resulted in the hybrids studied here would have included both favorable and unfavorable conditions. Similarly, the ERA series, resulting from a breeding program in the USA, also shows essentially parallel yield progress in growing areas with or without irrigation^7,16^, although the latter is associated with low yield^58^. In the ERA program, the released varieties bred for drought tolerance showed similar yield increases in dry and wet experiments^16^. Similar results were also found in Argentina with another set of varieties, with a rate of genetic progress similar to that reported here^14^. A common rate of genetic progress was observed in water deficit and well-watered conditions and at different fertilization rates, although observed mean yields ranged from 5 to 11 t ha^−1^ in worst vs best conditions^59^. Interestingly, these authors observed an increased number of ovaries and ear growth rate but a stable vegetative shoot growth rate, consistent with results presented here^60^. Hence, our conclusion is likely valid for the temperate maize growing area. It remains to be tested in subtropical and tropical regions where maize is grown.

The interpretation we propose here is that genetic variabilities for stomatal control, for parsimonious leaf growth under water deficit, for water-use efficiency, or for more stable yield exist in the breeding pool for temperate dent maize, but that they were not exploited by the breeding processes over the last 60 years. This is backed by the examples in Figs. 8 and 9. The yield QTLs whose effects largely vary with environmental scenarios were neither fixed by the selection nor were the related adaptive traits whose effects on yield can be either positive or negative depending on the environmental context. Because sensitivity traits have different effects on yield in dry vs wet years^29^, allelic frequencies in genomic regions governing these traits have likely oscillated with year-to-year climatic variability during the breeding process, without an overall trend^54^. Hence, sensitivity traits have not been exploited thus far, suggesting an untapped reservoir of favorable alleles.

However, it remains to be demonstrated that exploiting this reservoir will bring comparative advantages for yield. Indeed, it was stated that, if this pool was not exploited but a substantial yield improvement was still observed, breeding based on yield (directly or via genomic selection) may well remain the most promising strategy^15^. While recognizing that this statement is fair, we propose two arguments against it. Firstly, climate change will bring increasingly frequent events of severe drought or high temperatures. Breeding for the resilience to such events requires exploration of physiological adaptive traits, which may have neutral or slightly negative effects on yield in favorable conditions but allow new varieties to cope with extreme events (such a trade-off of yield potential vs risk is indeed widely accepted for crop cycle duration in a given growing area^5^). Because the climate is rapidly changing and breeding for new varieties takes years, it would probably be safe to explore this avenue. Secondly, the constitutive traits associated with yield progress in this study may reach a limit for future genetic progress, because the duration of the vegetative period and the distribution of foliage in the canopy cannot be indefinitely optimized. The high and increasing frequencies of positive alleles at some QTLs for these traits suggest that a limit might be reached in a near future.

Advances in phenotyping and novel breeding methods may help exploit the genetic variability of physiological adaptive traits. (i) The most obvious of those would be to select for yield stability in addition to high yield. However, yield stability cannot be considered as an objective per se because it may select for the lower yield. (ii) A major emphasis on suboptimal environments may be reached by systematically exploring the genotype × environment × management landscape^61^. This would allow one to refine selection for each environmental scenario, regardless of the actual location of an experiment in considered regions. (iii) Using the genetic variability of response curves of yield to temperature, evaporative demand, and soil water status may also be achieved with big data strategies that integrate phenomics, modeling, and genomic prediction^29,58^. Selection would then be carried out, in addition to yield itself, on the response of yield to environmental conditions clustered into growth-oriented vs conservative strategies. This would allow one to fine-tune hybrids for different environmental scenarios and present farmers with a larger offer, for them to choose the most appropriate hybrids based on their knowledge of each field and on their attitude towards risk^23^.

## Methods

### Genetic material

The genetic progress panel used in this study consisted of 66 highly successful commercial hybrids (Supplementary Table 1) released on the European market from 1950 to 2015. Experiments in 2010-2013 were performed with hybrids from 1950 to 2010, while further experiments included hybrids released after 2010. Three hybrids considered in early experiments could not be continued because the seed was not available anymore. Some phenotypic installations were able to only include a sub panel, which was sampled for an optimum representation of breeding generations (Supplementary Table 2). The presence of hybrids in all experiments is presented in Supplementary Table 1. The panel was designed to show a limited range of maturity classes, from mid-early (FAO 280) to mid-late (FAO 490) covering the largest growing area in Europe. Sixty of the hybrids were genotyped with a 600k Axiom Affymetrix array^62^. Data can be found and downloaded at 10.15454/KLD0GH ‘genotypic dataset’. After quality control, 465,621 polymorphic SNPs were retained for analysis (excluding SNPs with minor allele frequency lower than 0.05 and/or missing value for more than 20% of hybrids). Missing values were otherwise imputed^63^. All physical positions referred to hereafter are based on the B73 reference genome^64^.

We tested the representativeness of the above panel of varieties (named ‘genetic progress panel’ below) by comparing it with other two panels. First, we considered a series of 96 lines with expired Plant Variety Protection, known as ex-PVPs. These represent the founding material and germplasm pool for maize breeding from 1970 to 2000 in America and Europe^65,66^, for which genotypic information is available based on a set of 29,296 public SNP markers^67^. Second, we considered a panel of hybrids presented elsewhere^21,28^ resulting from the cross of a common flint parent (UH007) with 250 dent lines that maximized the diversity of temperate dent germplasm while keeping a restricted flowering window (called diversity panel hereafter), with the same range of earliness as the genetic progress panel. The parental lines of the diversity panel were also genotyped with the 600k Affymetrix array, as well as 500k markers obtained by genotyping-by-sequencing^28^. It was phenotyped in four experiments in a phenotyping platform^30–32^ and in 29 field experiments^21,29^ carried out in the same regions as those presented in Fig. 1a. We compared the three panels using a principal component analysis (PCA, R softare v3.6) based on common markers. The positions of founder lines of major heterotic groups were identified on the biplot (Supplementary Fig. 1). Finally, we performed another PCA on the genetic progress panel, with the 465,621 polymorphic SNPs, and analyzed the correlation of the first axis of the PCA with the year of release of corresponding varieties (R^2^ = 0.70, Supplementary Fig. 2). This regression was used in the genomic analysis below. The genome origin of each variety was determined via STRUCTURE^68^, using prior information on genetic groups (Iodent, Lancaster, Stiff Stalk) to estimate admixture proportions^28^. Pairwise identity-by-state (IBS) estimates^69^ ranged from 0.65 to 0.99 (av. 0.72), compared to IBS of 0.58–0.95 (av 0.66)^29^ in the diversity panel. Nei’s genetic diversity index^70^ was stable across old, intermediate, and recent groups of hybrids, thereby suggesting the maintenance of allelic diversity across years of release^71^.

### Yield and environmental conditions in Europe

We tested the representativeness of the experiments presented here based on the information^5^ collected over 35 years in 59 locations representative of the European maize growing area and of typical soil types of these regions, representing the ten European countries with the highest maize growing. Briefly, soil data were obtained from the JRC European Soil Commission database and from the Crop Growth Monitoring System. Meteorological data represent 35 years of daily weather (1975–2010) obtained from (i) the AGRI4CAST database of the JRC or the INRAE CLIMATIK databases. Sowing dates were simulated in each field as the first day from February to May in which the frequency of frost was <5% in the following ten days. We showed that these rules captured the variability of sowing dates in European databases^5^. Two watering regimes were simulated in each site, either rainfed or watered every seventh day with a water volume equaling the difference between reference evapotranspiration and rainfall. Plant density was adjusted in each country based on the JRC database. Nitrogen supply at sowing was calculated as 70% of nitrogen needed to reach the maximum yield of the optimum genotype with the optimum sowing date. Yields presented in Fig. 1 were simulated based on the information on climate and management practices presented above, over the 59 × 35 combinations of location and year^5^. For that, we used a modified version of the APSIM model^72^ parametrized for the B73×UH007 hybrid^73^.

### Platform experiments

The seven platform experiments presented here are synthesized in Supplementary Table 2, and the hybrids involved in each of them are presented in Supplementary Table 1. The phenotypic variables considered in each experiment are presented in a spreadsheet (Supplementary data 11) in which each sheet corresponds to one experiment and presents in detail the traits measured in this experiment with their entity (e.g., ear or plant), characteristic (e.g., area), methods, unit, and phenological stages at which each measurement was taken. This file also presents the mapping of these terms onto public ontologies. Each of these sheets is presented again as a csv file in an experiment-specific directory of the dataset 10.15454/KLD0GH, together with complements of methods and the datafile from which all values can be downloaded.

Three experiments were carried out at the Phenoarch platform^26^ https://www6.montpellier.inrae.fr/lepse/Plateformes-de-phenotypage/Montpellier-Plant-Phenotyping-Platforms-M3P/PhenoArch. They followed an alpha-lattice design with seven, four, three and three plants per genotype in experiments a, c, and d, respectively (Supplementary Table 2). Experiments involved treatments with contrasting soil water status and air temperature. Two levels of soil water content were imposed, either retention capacity (WW, mean soil water potential of −0.05 MPa) or water deficit (WD) in which soil water potential was individually maintained at −0.4 MPa for each pot^30^. The greenhouse temperature was 25 ± 3 °C and 18 ± 1 °C during day and night. In Exp a, the high-temperature treatment (HT) involved transferring four plants per hybrid to a greenhouse at 33 ± 3 and 21 ± 1 °C during day and night, from the 14th visible leaf stage until flowering time. Air temperature and humidity were measured at six positions on the platform every 15 min^30^. The temperature of the meristematic zone was measured with fine thermocouples in either twenty (Exp. a) or six (other experiments) plants per experiment. Daily incident photosynthetic photon flux density (PPFD) over each plant was estimated by combining a 2D map of light transmission in the greenhouse with the outside PPFD averaged every 15 min^26^. Supplemental light was provided either during daytime when external solar radiation was below 300 W m^−2^ or to extend the photoperiod (14 h) with 0.4 lamps m^−2^. The number of visible and ligulated leaves of each plant was recorded every week. This information was used in a model of progression of leaf stage for each hybrid as a function of thermal time, used to predict phenological stages for all hybrids in all field experiments^29^.

Architectural traits were calculated from 3D reconstructions of each plant in the platform (Exp. a), based on RGB (2056 × 2454) images taken from thirteen views (twelve side views from 30° rotational difference and one top view) captured daily for each plant during the night. The distribution of leaf area along the stem was calculated from 3D reconstructions^44^. An index, rh_PAD_, represented the point in this distribution (from the top of the canopy, relative to total canopy height) where half of the cumulative leaf area was observed^44^. The radiation intercepted by the canopy layer including the ear was calculated in simulated canopies repeating each plant in the platform, at densities of 6, 8, or 10 plants m^−2^ ^44^. Total leaf area and shoot biovolume (transformed into biomass via periodic measurements of plant water content) were obtained every day from 3D reconstructions (Exp. a) or estimated by regression with pixel number in Exp. c and d^30^. Radiation use efficiency (RUE) was calculated as the ratio of biomass to intercepted light, with the latter calculated from 3D plant reconstructions^44^.

Reproductive development was studied in Exp. a, c and d. Four plants per hybrid were sampled on the first day of pollen shedding, individually recorded for each plant. Ears were dissected to measure (i) silk length (from ovary insertion to tip) and the number of emerged silks^42^ (silks are modified styles of each ovary that collect pollen) and (ii) the number of ovary cohorts per ear^38^ (rings of ovaries or grains initiated at a common date, localized at a common distance from the ear base). Raw trait values were corrected for spatial disturbances by fitting a mixed model to daily measurements (R package *SpATS*^74,75^) with a fixed term for genotype and random effects for rows and columns as well as a smooth surface defined on row and column coordinates. Generalized heritabilities were calculated daily with the same R package, using the same model but with genotypes included as a random term^74,75^. Genotypic predictions for traits at individual time points, *t*, were obtained from a generalized additive model fitted to the spatially adjusted daily measurements, ${\widetilde{y}}_{i,k}\left(t\right)$, for each plant *k* of genotype *i*

1 $${\widetilde{y}}_{i,k}\left(t\right)={\alpha }_{i}+{f}_{i}\left(t\right)+{\epsilon }_{i,k}\left(t\right),{\epsilon }_{i,k}\left(t\right) \sim N\left(0,{\sigma }^{2}\right)$$

where ${\alpha }_{i}$ is a genotype-specific intercept, ${f}_{i}\left(t\right)$ is a genotype-specific thin plate regression spline function on time, and ${\epsilon }_{i,k}\left(t\right)$ is a random error term. Model (1) was fitted with the gam function of the *mgcv* R package by REML. Variance components were obtained by treating smooth terms as random effects

Three series of experiments were performed in the Phenodyn platform^76^, https://www6.montpellier.inrae.fr/lepse/Plateformes-de-phenotypage/Montpellier-Plant-Phenotyping-Platforms-M3P. A completely randomized design was used involving three replicates per hybrid. Leaf elongation rate (LER), expressed per equivalent days at 20 °C^77^, was measured with a 15 min temporal definition on the sixth appeared leaf^51^. Three treatments were imposed, well-watered (soil water potential ranging from 0 to −0.05 MPa), water deficit in which soil was left to dehydrate from −0.05 to −1.2 MPa, and high evaporative demand. The latter treatment involved transferring well-watered plants to a growth chamber in which steps of air vapor pressure deficit (VPD_air_) were imposed from 0.8 to 3 kPa^51^. Air temperature and relative humidity were measured at plant level with nine sensors^51^, the temperature of the meristematic zone was measured as above in six plants, photosynthetic photon flux density (PPFD) was measured using nine sensors^51^. The transpiration rate was calculated as in the previous work^51^. The genotypic sensitivities to soil water potential and VPD were estimated as the slopes of the regression of leaf elongation rate on soil water potential or VPD_air_^78^. In Exp. g, transpiration rates were scaled by individual plant leaf area, and smoothed by Nadaraya-Watson kernel regression with an optimal span of 0.25, using the R package *stats*. Genotypic estimates of transpiration rate were then calculated for each time-point with a mixed model adjusting for spatial effects as above. They were used for the calculation of stomatal conductance every 15th minute by inversion of the Pennman-Monteith equation^30^. Generalized heritabilities were calculated as above.

One experiment (Exp. h) was carried out in the RootPhAir platform (https://uclouvain.be/en/research-institutes/eli/elia/rootphair.html), with a completely randomized design involving 30 hybrids and 30 replicates per hybrid (Supplementary Table 2). The greenhouse temperature was 23 ± 3 °C and 16 ± 2 °C during day and night. Supplementary LED lighting was used to obtain a 16:8 photoperiod. Seeds were pre-germinated on vertical filter paper and transferred to the aeroponics platform. The latter allows roots to grow at a rate only constrained by assimilating availability and temperature. Shoot and root dry and fresh weights were measured after 2 weeks. Genotypic means were corrected for spatial effects as above.

### Field experiments

The 30 field experiments presented here are synthesized in Supplementary Table 2. The phenotypic and environmental variables considered in each experiment are presented in a spreadsheet (Supplementary data 1) in which each sheet corresponds to one experiment, as presented above for experiments in controlled conditions. Each of these sheets is presented again as a csv file in an experiment-specific directory of the dataset 10.15454/KLD0GH, together with complements of methods and the datafile from which all values can be downloaded.

One intense field experiment (Exp. k) tested the effect of plant density in the Phenofield platorm^79^. Plants were sown at densities of either 7 or 9 plants m^−2^, either irrigated or with a water deficit imposed by rain-out shelters. The experiment followed an alpha-lattice design with three replicates of two-rows plots, 6 m long. Light, air temperature, relative humidity (*RH*), and wind speed were measured every hour with an on-field weather station. Soil water potential was measured every day with tensiometers at 30, 60, and 90 cm depths with three replicates per treatment. Plots were mechanically harvested, then grain yield was scaled to 15% moisture content after estimation of grain moisture at harvest. Individual grain weight was measured and used to calculate grain number per square meter from grain yield. The proportion of the green fraction was calculated from images taken horizontally above the canopy^80^.

Two intensive field experiments (Exp i, j) were carried out in the DIAPHEN platform of Mauguio, near Montpellier. Experiments followed an alpha-lattice design with three replicates of four-row plots. The light was measured with PPFD sensors at 2 m height in the immediate vicinity of experimental fields. Air temperature and *RH* were measured in ventilated shelters for calculation of VPD_air_. Soil water potential was measured every day with tensiometers at 30, 60, and 90 cm depths in watered and rainfed microplots with three and two replicates, respectively. Each day was characterized by the VPD_air_ for the three hours in the afternoon during which VPD_air_ was maximum, referred to as VPD_max_. The progression of the crop cycle was expressed in d_20 °C_ after emergence. Visible leaf number was recorded twice during the vegetative phase for 10 plants per plot for all field experiments. The number of fertile ears per plant was measured at harvest. The number of cohorts and the number of grains per cohort were manually counted from ears harvested from ten plants per plot. Silk length at silking was measured on ten plants per plot. Traits were averaged per plot and spatially adjusted as in the multi-site experiment below. In experiment j, images were taken from a hexacopter Atechsys (http://atechsys.fr/) that carried a six-channel multispectral camera (Hi-Phen modèle V3 AirPhen 6 bands (450–532–568–675–730–850). The leaf area index was calculated as the projected leaf area per unit ground surface area, generated by inverting the radiative transfer model PROSAIL^81^. The fraction of intercepted photosynthetically active radiation was calculated from RGB images. The mean leaf angle was calculated by inverting the radiative transfer model PROSAIL^81^. Other variables calculated from these images are presented in the datafile 10.15454/KLD0GH but not used in this study.

A multi-site experiment involved 26 field experiments (each defined as a combination of location × year × water regime, Exp. i, j, l, m, i.e., including intensive fields i and j presented above) between 2010 and 2017 in 16 European locations spread along a climatic transect for temperature and evaporative demand in both rainfed and irrigated conditions (Fig. 1a, Supplementary Table 2). Experiments followed an alpha-lattice design with two replicates of four-row plots in well-watered conditions, three replicates in rainfed conditions. The light was measured with PPFD sensors at 2 m height in the immediate vicinity of experimental fields. Air temperature and RH were measured in ventilated shelters for calculation of VPD_air_. Soil water potential was measured every day with tensiometers at 30, 60, and 90 cm depths in watered and rainfed microplots with three and two replicates, respectively. Each day was characterized by the VPD_air_ for the 3 h in the afternoon during which VPD_air_ was maximum, referred to as VPD_max_. Reference evapotranspiration (ET_0_) was calculated based on the Penman-Monteith equation as revised in the FAO-56 estimation^82^. The progression of the crop cycle in each site was expressed in d_20 °C_ after emergence. The amount of light intercepted by plants was simulated for each experiment using a modified APSIM crop model^73^ for the standard hybrid B73xUH007 under well-watered conditions but parametrized with the mean final leaf number of the panel^73^. As such, the amount of light intercepted is akin to an environmental variable. Visible leaf number was recorded twice during the vegetative phase for 10 plants per plot for all field experiments, to check the model established based on experiments in PhenoArch. Sowing, harvesting and emergence dates were recorded for each experiment. Anthesis and silking dates were recorded for each plot when half the plants reached these stages. The number of fertile ears per plant was measured at harvest. The number of grain cohorts was calculated in each experiment by dividing grain number by the mean ear number per plant (as measured in each experiment) and by the number of grains per cohort measured in platform and intensive field experiments, very stable for each hybrid (Supplementary Fig. 5). The two central lines of each plot were mechanically harvested, and grain yield was scaled to 15% moisture content. Individual grain weight was measured and used to calculate grain number per m^2^ from grain yield. BLUEs were calculated, for grain yield components and flowering traits, by fitting a mixed model that included a fixed term for genotype, row and column as random terms and a smooth surface, using the R package *SpATS*^74,75^. Generalized heritabilities were then calculated with genotype included as a random term^74,75^.

The above field experiments were clustered into six environmental scenarios based on light, temperature, VPD_max_, and soil water status. A principal component analysis was performed on cumulated intercepted light and daily minimum and maximum temperatures over the vegetative, flowering and grain-filling phases defined as earlier^29^, followed by clustering of experiments based on partitioning around medoids. Three consistent temperature scenarios were identified, namely cool, warm and hot, with mean minimum temperatures of 15, 17, and 18 °C and mean maximum temperature of 25, 28, and 30 °C, respectively. The Hot scenario was also characterized by a longer period during which temperatures were at high values (Fig. 1e). Soil water conditions, clustered over the same phases, resulted in two scenarios (Fig. 1f), namely well-watered (mean soil water potential during the vegetative and flowering phases above −0.1 MPa) and water deficit (mean soil water deficit below −0.1 MPa, see time courses in Fig. 1f). Evapotranspiration was simulated with the APSIM crop growth model^72^, which simulates plant growth, transpiration and soil evaporation every day, based on environmental data and parametrized for the B73×UH007 hybrid but with final leaf number, phyllochron and ligulochron set to the mean genotypic estimate over four generations of hybrids^73^. Calculations at the base of Supplementary Fig. 13 follow those in ref. ^52^. Briefly, yields measured in each experiment were plotted against evapotranspiration simulated as above in the same field for each studied hybrid. Hybrids were then clustered by year of release.

### Genetic gain, Bayesian networks, and genotype × environment interaction

Genetic gain for a trait was defined by the slope of the linear regression of the trait value on the year of release in the genetic progress panel. Genotypic means for traits were obtained for each environmental scenario or experimental treatment with a linear mixed model including a fixed term for hybrids and a random term for experiments (R package *lme4*). A Bayesian network was constructed (R package *bnlearn*) to evaluate the conditional dependencies between year of release, measured phenotypic traits and grain number. For each environmental scenario, acyclic-directed Bayesian networks were fitted to scenario-specific genotypic means of grain number and phenology, together with estimates for traits measured in phenotyping platforms, centered and scaled prior to analysis. Straightforward assumptions were made in the analysis. Firstly, the year of release was assumed to be a driver for the considered traits (i.e., no arc from traits to a year of release). Secondly, conditional dependencies between phenotypic traits followed the temporal order of plant development^25^, i.e., traits determined during the vegetative and flowering periods were assumed as not conditionally dependent on grain number, which is fixed at the beginning of the grain-filling period. Network learning was conducted with the TABU algorithm combined with the Bayesian gaussian equivalent score (BGE), which assumes non-informative flat priors over both the space of network structures and of parameters^83^. Scenario-specific models were fitted to the corresponding genotypic means of traits for 500 bootstrap samples, and subsequently averaged across all bootstrap iterations^83,84^. Model averaging was based on an empirical test, whereby significant arcs were maintained^83,84^. Scenario-specific predictive ability of averaged models was then evaluated by ten times repeated five-fold cross-validation. Correlation between predicted and observed values was 0.7 on average across traits and scenarios. A synthesis is presented in Fig. 3a representing the arrows that were consensus across scenarios.

We then further investigated the rate of yield progress in various linear models for grain number per square meter^29^, i.e., the dependence of grain number on the year of release. To model grain numbers across multiple experiments grouped into environmental scenarios, the linear models used contained terms for genetic main effects (including generation of selection), and additional terms for environmental main effects and genotype-by-environment interaction. The inclusion of terms was tested by ANOVA-based forward selection and backward elimination with the R package *stats (R Core Team, 2020)*. The grain number was modeled as a response to a genotypic main effect, an environmental main effect, and a residual term that included the genotype by environment interaction. The linear model was fitted with the R package *stats*^85^. Genotypic and environmental effects were further partitioned by including a fixed genotypic covariate term for the year of release (${G}_{{{{{{{\mathrm{year}}}}}}}}$) and a fixed factor representing environmental scenarios *(Scen)*, respectively. After the introduction of a genotypic covariate and an environmental scenario effect, a residual genotypic main effect (${G}_{{{{{{{\mathrm{res}}}}}}}}$) was included alongside a residual environmental effect “experiment within scenarios” (${E}_{{{{{{{\mathrm{res}}}}}}}}$). The interaction between genotypes and environment was partitioned into a part due to *G*_*year*_ *× Scen* (GEI_year_ in Fig. 3b) and a term:

2 $${GN}=\,\mu +({G}_{{{{{{{\mathrm{year}}}}}}}}+\,{G}_{{{{{{{\mathrm{res}}}}}}}})+({{{{{{\mathrm{Scen}}}}}}}+\,{E}_{{{{{{{\mathrm{res}}}}}}}})+({G}_{{{{{{{\mathrm{year}}}}}}}}\times \,{{{{{{\mathrm{Scen}}}}}}})+\,\varepsilon$$

where *µ* is the intercept. We then investigated the relative contributions of key categories of traits -phenology, reproductive and architecture- to the variance explained for grain number. This resulted in a further partition of genotypic main effects by the duration of the vegetative phase (*Veg*), number of extruded silks *(Silks)*, and plant architecture (*rh*_*PAD*_). In addition, *G*_*year*_, was again included to assess the extent to which genetic gain was captured by those three covariates. Finally, a residual genotypic main effect was added. For the environmental main effect, again we included the partitioning of trial main effects into scenarios (*Scen*) and experiments within scenarios (*E*_*res*_). The interaction between the genotypic covariate year of release and scenario was also included (${G}_{{{{{{{\mathrm{year}}}}}}}}\times {{{{{{\mathrm{Scen}}}}}}}$).

3 $${GN}= 	\,\mu +({{{{{{\mathrm{Veg}}}}}}}+{{{{{{\mathrm{Silk}}}}}}}+{rh}{{{{{{\mathrm{pad}}}}}}}+\,{G}_{{{{{{{\mathrm{year}}}}}}}}+\,{G}_{{{{{{{\mathrm{res}}}}}}}})+\,({{{{{{\mathrm{Scen}}}}}}}+\,{E}_{{{{{{{\mathrm{res}}}}}}}})\\ 	 +\,({G}_{{{{{{{\mathrm{year}}}}}}}}\times \,{{{{{{\mathrm{Scen}}}}}}})+\,\varepsilon$$

Finally, we dissected the interaction of year and scenario by factorial regression on environmental indices:

4 $${GN}=\,\mu +\left({G}_{{{{{{{\mathrm{year}}}}}}}}+\,{G}_{{{{{{{\mathrm{res}}}}}}}}\right)+\,\left({{{{{{\mathrm{Scen}}}}}}}+\,{E}_{{{{{{{\mathrm{res}}}}}}}}\right)+\,{\beta }_{1}\varPsi +{\beta }_{2}R{{{{{{\mathrm{int}}}}}}}+\varepsilon$$

where *β*_1_ is the genotypic sensitivity to mean soil water potential at flowering, Ψ, and *β*_2_ is the genotypic response to the intercepted radiation during the vegetative phase, *R*_int_. The environmental indices were included sequentially by step-wise model selection, as described above.

### Genome-wide scans of regions under selection and multi-trait meta-analysis

We performed genome-wide scans to identify regions under selection (RUS), based on two independent methods:

- We identified loci with robust differentiation between the 22 oldest and 22 most recent hybrids of the genetic progress panel (Supplementary Table 1) using a Bayesian *X*^*T*^*X* statistic that minimizes false associations by accounting for genome-wide covariance in allele frequencies^53^. *X*^*T*^*X* identifies loci with differences in group-wise allele frequencies that strongly depart from genome-wide differences arising from population structure, putatively identifying loci underlying changes due to selection. Bayenv 2.0 was run using default settings, with a random subset of 8946 SNPs pruned according to local LD^86^ to compute the covariance matrix. As recommended by the authors, markers exceeding the 99.95th quantile of *X*^*T*^*X* values were considered RUS.
- We carried out a regression between the year of release and allele counts for the alternative SNP to the B73 reference genome (0, 1, or 2 whether the considered allele is homozygous for B73 allele, heterozygous or homozigous for the alternative allele, respectively) over the whole panel (Supplementary Fig. 2) while controlling for the relatedness structure using a genomic relationship matrix (GCTA software: flag --mlma^87^. SNPs were ranked based on the −log10(*p*-value) for the regression coefficient. A threshold of 3.5 was used for identifying SNPs whose allelic values changed most with year of release, and classified as belonging to RUS (Supplementary Fig 2c).

Finally, we tested for the enrichment of colocalization between RUS and QTLs for studied traits, compared to proportions expected for an equal number of random genomic regions having the same physical size as the studied QTL intervals, using a permutation-based approach. We sampled, 100,000 times, random regions from the genome with matching numbers and physical lengths compared with the studied QTLs while keeping the RUS positions fixed. At each iteration, we counted the number of random regions overlapping with RUS. A null distribution was built based on these 100,000 values, to which the observed number of overlaps was compared via a Chi2 test (function *permp* in the R package *statmod*^88^). Because published QTLs were positioned on the V2 version of the genome^21,30–32^, the whole study was performed on this version. Colocalization of QTLs was tested for: (i) QTLs for days to anthesis, days to silking and final leaf number identified in the panel of lines^33^, (ii) eQTLs for the florigens ZCN8, ZCN12, and ZCN7 in the same panel^33^, (iii) QTLs for maximal stomatal conductance and difference in biomass between well-watered and water deficit treatments, measured in a series of experiments in PhenoArch^30^ and calculated in the hybrid panel^28^, (iv) QTLs for radiation interception efficiency (RIE) and vertical distribution of leaf area measured in PhenoArch and calculated as in ref. ^44^ in the hybrid panel, (v) QTLs for the slope of the response curve of leaf elongation rate to soil water potential measured in PhenoArch in the hybrid panel, (vi) QTLs for grain yield in the hybrid panel, observed in fields classified in scenarios with high temperature or water deficit, but not in well-watered conditions^21^ (vi) QTLs for the slope of the responses of grain number to soil water potential, night temperature and light intensity^29^ in the same panel.

### Calculation of allelic effects of QTLs in the 24 fields of the multi-site experiment and of allelic frequencies at these QTLs in the 22 most ancient and 22 most recent hybrids of the panel

We considered six QTLs identified previously in the diversity panel, whose effects depend on the environmental scenarios defined as a function of soil water status and air temperature (Fig. 9). We ascribed each of the 24 fields of the multi-site experiment to the scenarios as defined in Millet et al.^21^ using measured environmental conditions in each field of the multi-site experiment (Exp i,j,l,m as in Supplementary Table 2). For each QTL, the allelic effect estimated in the previous study was assigned to each field as a function of its environmental scenario. The frequency of the B73 allele was calculated for the peak marker at each QTL^21^ in the 22 most ancient and 22 most recent hybrids of the genetic progress panel. Frequencies presented in Fig. 9d are those of the allele that provides positive effect in the most favorable condition for yield, namely cool and well-watered.

### Reporting summary

Further information on research design is available in the Nature Research Reporting Summary linked to this article.

## Supplementary information

Supplementary Information

Description of Additional Supplementary Files

Supplementary Dataset 1

Supplementary Dataset 2

Supplementary Dataset 3

Supplementary Dataset 4

Supplementary Dataset 5

Supplementary Dataset 6

Supplementary Dataset 7

Supplementary Dataset 8

Supplementary Dataset 9

Supplementary Dataset 10

Supplementary Dataset 11

Reporting Summary

## Data Availability

The Phenotypic and genotypic data generated in this study have been deposited in the DATA.INRAE database under accession code 10.15454/KLD0GH. They are presented with necessary metadata and explanation of the dataset.
