# Supplementary material for: Physiological adaptive traits are a potential allele reservoir for maize genetic progress under challenging conditions
Source: Nat Commun. 2022 Jun 9;13:3225. doi: 10.1038/s41467-022-30872-w (PMC9184527; doi:10.1038/s41467-022-30872-w)
Supplement: Supplementary file 2 — Description of Additional Supplementary Files [file 41467_2022_30872_MOESM2_ESM.pdf]

File name: Supplementary data 1
Description: Raw data for figure 1

File name: Supplementary data 2
Description: Raw data for figure 2

File name: Supplementary data 3
Description: Raw data for figure 3

File name: Supplementary data 4
Description: Raw data for figure 4

File name: Supplementary data 5
Description: Raw data for figure 5

File name: Supplementary data 6
Description: Raw data for figure 6

File name: Supplementary data 7
Description: Raw data for figure 7

File name: Supplementary data 8
Description: Raw data for figure 9

File name: Supplementary data 9
Description: Raw data for Supplementary Fig. 9

File name: Supplementary data 10
Description: Raw data for Supplementary Fig. 13

File name: Supplementary data 11
Description: Overview of traits and environmental variables measured in each experiment, with trait entity, trait characteristic, methods and units, and the mapping of those onto public ontologies

Supplementary Fig. 1 | Principal component analysis based on a set of 29296 public common markers, for the 66 varieties analysed in this study (GP, squares), compared with 98 patent-expired maize lines in the public domain (black dots, exPVPs, Plant Variety Protection) and with 250 lines of a dent diversity panel (blue dots). For genetic progress varieties: red, released earlier than 1980; orange, 1980-2000; green, later than 2000. The positions of founder lines of major heterotic groups are identified on the biplot. The first two axes are presented in panel a, axes 3 and 4 in panel b. Number in parentheses in the axes legends represent the proportion of variability captured by each axis. Source data in https://doi.org/10.15454/KLD0GH /genotypic dataset for the genetic progress panel, in Negro et al 2019 for the diversity panel and Coffman et al (2020) for exPVPs

Supplementary Fig. 2 | Diversity analysis of the panel of genetic progress, and relationship with year of release. a, Principal Component Analysis (PCA) based on 460k SNPs. b, The pearson correlation between the coordinates of hybrids on the first principal component axis (PC1) of the PCA and year of release. c, Manhattan plot based on the $-\log_{10}$ (p-value) of the regression between the year of release and allelic values at the 460k SNPs, after correction for population structure. In b, the grey zone is the 0.95 interval of confidence of the fitted regression. In c, the red line indicates the threshold identifying the SNPs with values that changed most with year of release. Source data in https://doi.org/10.15454/KLD0GH /genotypic dataset

Supplementary Fig. 3 | Change with year of release of grain yield for two plant densities. Experiment k: 7 and 9 plants m^{-2}, with two water regimes (water deficit or well-watered: water potential higher or lower than –0.1 MPa throughout the plant cycle). Each dot is the genotypic BLUE per hybrid. Regression lines are drawn when significant. In WW, p values =2.1 10^{-5} and 3.4 10^{-6}. In WD pvalues = 2.2 10^{-6} and 7.0 10^{-5}. Source data in https://doi.org/10.15454/KLD0GH/phenomic dataset/exp_k_Phenofield_2018/exp_k_Phenofield_2018_data, together with metadata (trait entity, trait characteristic, method, unit, phenological stage) and the mapping of this information onto public ontologies

Supplementary Fig. 4 | Changes in grain number (a-c) and individual grain weight (d-f) with year of release and relation between grain yield and grain number (g-i). One panel per temperature scenario of experiments (cool, warm and hot, respectively), with the two water scenarios in each panel. Experiments i, j, l and m. a-f, One dot per genotype x scenario (BLUEs). g-j, One dot per genotype x experiments (BLUEs). Pvalue of regressions for individual grain weight in warmWW and CoolWD: 0.008 and 0.012, respectively. All other pvalues lower than 10^{-5}. Source data in https://doi.org/10.15454/KLD0GH

phenomic dataset /Exp_lm_multisite/phenotypic data and indicators /Expijlm_BluesPerScenario_data, together with metadata (trait entity, trait characteristic,

Supplementary fig. 5 | Boxplots of grain yield and grain number in the panel, compared with genetic progress, in each environmental scenario. Green and orange arrows, median of the phenotypic value of the 22 most recent and 22 most ancient hybrids, respectively. Phenotypic values taken into account are the BLUES for each hybrid (60 hybrids for all scenarios except Cool_WW (56), and cool_WD (57). Experiments i, j, l and m as in supplementary Table 2. Bold lines in boxes, median; boxes, 25 and 75 percentiles; vertical lines, 25 and 75 percentiles multiplied by 1.5 of the interquartile range (25-75). Source data in https://doi.org/10.15454/KLD0GH /phenomic dataset /Exp_lm_multisite/phenotypic data and indicators /Expijlm_BluesPerScenario_data, together with metadata (trait entity, trait characteristic, method, unit, phenological stage) and the mapping of this information onto public ontologies.

Supplementary fig. 6 | Boxplots of reproductive traits and drought responsive traits in the panel, compared with genetic progress. Green and orange arrows, median of the phenotypic value of the 22 most recent and 22 most ancient hybrids, respectively. Phenotypic values taken into account are the BLUES for each hybrid (60 hybrids for all scenarios and traits in panel a, 56 hybrids in panel b left, 48 hybrids in panel b right). Experiments a for reproductive traits, f and h for leaf elongation rate and stomatal conductance, respectively. Stomatal conductance measured at PPFDs between 800 and 1200 µmol m^{-2} s^{-1} for whole plants. Bold lines in boxes, median; boxes, 25 and 75 percentiles; vertical lines, 25 and 75 percentiles multiplied by 1.5 of the interquartile range (25-75). Source data in https://doi.org/10.15454/KLD0GH /phenomic dataset / exp_a_PhenoArch_ZA17/exp_a_PhenoArch_ZA17_data for panel a, exp_g_ArchDyn_ZA20/exp_g_ArchDyn_ZA20_data for panel b left and exp_ef_Phenodyn/exp_ef_Phenodyn_data for panel b right, together with metadata (trait entity, trait characteristic, method, unit, phenological stage) and the mapping of this information onto public ontologies

Supplementary Fig. 7 | Change with year of release of the number of ovaries or grains per cohort. Blue and red symbols, well watered (soil water potential higher than -0.1 MPa) and water deficient plants. Blue triangles in a, well watered plants with heat stress Orange symbols in d, intermediate soil water deficit. Each point, BLUE corresponding to one hybrid. Regression lines are presented here for better intuition but are not significant. Pvalues of regressions: 0.9, 0.9 and 0.8 in a, 0.6 in b, 0.93 in c, 0.31 and 0.32 in d. Source data in https://doi.org/10.15454/KLD0GH /phenomic dataset / exp_a_PhenoArch_ZA17/exp_a_PhenoArch_ZA17_data, for panel a, exp_c_PhenoArch_ZD13/exp_c_PhenoArch_ZD13_data, for panel b, exp_d_PhenoArch_ZE11/ exp_d_PhenoArch_ZE11_data, for panel c, and

exp_i_Diaphen2010/ exp_i_Diaphen2010 _data, for panel d, together with metadata (trait entity, trait characteristic, method, unit, phenological stage) and the mapping of this information onto public ontologies

Supplementary Fig. 8 | Boxplots of architectural traits, radiation use efficiency, leaf area an biomass in the panel, compared with genetic progress. Green and orange arrows, median of the phenotypic value of the 22 most recent and 22 most ancient hybrids, respectively. Phenotypic values taken into account are the BLUES for each hybrid. Experiment a as in supplementary Table 2. Bold lines in boxes, median; boxes, 25 and 75 percentiles; vertical lines, 25 and 75 percentiles multiplied by 1.5 of the interquartile range (25-75).Source data in https://doi.org/10.15454/KLD0GH /phenomic dataset / exp_a_PhenoArch_ZA17/exp_a_PhenoArch_ZA17_data

Supplementary Fig. 9 | Change with year of release of the response of grain number to the amount of incident light intercepted during the vegetative phase (a) and to the mean soil water potential during the flowering phase (b). Experiments i, j, l, m. One dot is the response of one hybrid (β) to the environmental variable, calculated in the factorial regression (equation (4) in the online method).). Regression lines are drawn when significant ($p<0.01$). Source data and pvalues in Supplementary data 9.

Supplementary Fig. 10 | Change with year of release of leaf area index (a,b) **and erectness** (c,d) **estimated by drone imaging, at vegetative stage (a,c) and flowering time** (b,d)**.** Three water regime are presented with well watered (blue) or water deficit (two intensities, red and orange). One dot per hybrid (BLUEs). Regression lines are drawn when significant ($p<0.01$). Pvalues of regressions: 0.003, 0.005 and 0.001 in a; 0.0007, 0.05 and 0.006 in c, 0.003, 0.005 and 0.001 in d. P values in $b<10^{-4}$. Source data in https://doi.org/10.15454/KLD0GH /phenomic dataset/exp_j_Diaphen2017/

exp_j_Diaphen2017_data, together with metadata (trait entity, trait characteristic, method, unit, phenological stage) and the mapping of this information onto public ontologies

Supplementary Fig. 11 | Change with year of release of leaf area at flowering time in platform and field experiments. a,b, Change with year of release of leaf area at flowering time, experiment a and d. c,d, Green fraction in images from above the canopy in exp k, for two plant densities (7 and 9 plants m^{-2}), and two water regimes. One dot per hybrid (BLUEs). Regression lines are drawn when significant (p<0.01). Pvalues of regressions, 2 10^{-4} and 6 10^{-5} in a; 0.6 in b; 0.21 and 0.37 in c, 0.44 and 0.28 in d. Source data in https://doi.org/10.15454/KLD0GH /phenomic dataset / exp_a_PhenoArch_ZA17/exp_a_PhenoArch_ZA17_data, for panel a, exp_d_PhenoArch_ZE11/ exp_d_PhenoArch_ZE11_data, for panel b, and

exp_k_Phenofield_2018/exp_k_Phenofield_2018_data for panels c and d, together with metadata (trait entity, trait characteristic, method, unit, phenological stage) and the mapping of this information onto public ontologies

Supplementary Fig. 12 | Change with year of release of (fresh) shoot biomass in platform experiments. Experiments a c and d. One dot per hybrid (BLUEs). Regression lines are drawn when significant (p<0.01). Pvalues of regressions, 0.003 and 0.001 in a; 0.7 in b; 0.04 in c. Source data in https://doi.org/10.15454/KLD0GH /phenomic dataset / exp_a_PhenoArch_ZA17/exp_a_PhenoArch_ZA17_data, for panel a, exp_c_PhenoArch_ZD13/ exp_c_PhenoArch_ZD13_data, for panel b, and

exp_d_PhenoArch_ZE11/ exp_d_PhenoArch_ZE11_data, for panel c, together with metadata (trait entity, trait characteristic, method, unit, phenological stage) and the mapping of this information onto public ontologies

Supplementary Fig. 13 | Relationship between grain yield and cumulated transpiration for each generation of hybrids represented by decade of release. a, The cumulated transpiration was calculated with the APSIM model and the grain yield was averaged per experiment (BLUEs). b, Relation between decade of release and the asymptote of the response curve for each decade (from a). Source data in Supplementary data 10.

Supplementary Fig 14 | Genomic analysis of regions under selection, compared with trait QTLs in chromosomes other than 8. Regions under selection (RUS) identified by (i) differentiation between the 22 most ancient and 22 most recent hybrids (top 0.05% Bayenv XtX method) or (ii) regression of allelic values with year of release (-log10 p-values > 3.5). b, c, d, QTLs involved in three categories of traits. b, QTLs of days to anthesis and eQTLs of the florigen ZCN12. c, QTLs involved in plant architecture (rhPAD and amount of light intercepted by the canopy layer where ears are located). d, QTLs of adaptive traits (stomatal conductance, sensitivity of leaf growth to water deficit, sensitivity of grain number to water deficit and yield QTLs that were observed in water deficit or high temperatures scenarios only. . Source data in https://doi.org/10.15454/KLD0GH /genotypic dataset for the physical map and in corresponding papers for QTLs
