# Supplementary material for: Physiological adaptive traits are a potential allele reservoir for maize genetic progress under challenging conditions
Source: Nat Commun. 2022 Jun 9;13:3225. doi: 10.1038/s41467-022-30872-w (PMC9184527; doi:10.1038/s41467-022-30872-w)
Supplement: Supplementary file 14 — Reporting Summary [file 41467_2022_30872_MOESM14_ESM.pdf]

nature research

Corresponding author(s): Francois Tardieu

Last updated by author(s): 4/22/2022

Reporting Summary

Nature Research wishes to improve the reproducibility of the work that we publish. This form provides structure for consistency and transparency in reporting. For further information on Nature Research policies, see our Editorial Policies and the Editorial Policy Checklist.

Statistics

For all statistical analyses, confirm that the following items are present in the figure legend, table legend, main text, or Methods section.

n/a	Confirmed	
☐	☒	The exact sample size (n) for each experimental group/condition, given as a discrete number and unit of measurement
☐	☒	A statement on whether measurements were taken from distinct samples or whether the same sample was measured repeatedly
☐	☒	The statistical test(s) used AND whether they are one- or two-sided<br>*Only common tests should be described solely by name; describe more complex techniques in the Methods section.*
☐	☒	A description of all covariates tested
☐	☒	A description of any assumptions or corrections, such as tests of normality and adjustment for multiple comparisons
☐	☒	A full description of the statistical parameters including central tendency (e.g. means) or other basic estimates (e.g. regression coefficient) AND variation (e.g. standard deviation) or associated estimates of uncertainty (e.g. confidence intervals)
☐	☒	For null hypothesis testing, the test statistic (e.g. F, t, r) with confidence intervals, effect sizes, degrees of freedom and P value noted<br>*Give P values as exact values whenever suitable.*
☐	☒	For Bayesian analysis, information on the choice of priors and Markov chain Monte Carlo settings
☐	☒	For hierarchical and complex designs, identification of the appropriate level for tests and full reporting of outcomes
☒	☐	Estimates of effect sizes (e.g. Cohen's d, Pearson's r), indicating how they were calculated

Our web collection on statistics for biologists contains articles on many of the points above.

Software and code

Policy information about availability of computer code

Data collection	Data collection in phenotyping platforms was performed using the PHIS information system. Neveu P., et al. New Phytologist 22, 588–601. Data organization followed principles presented in this paper and updated in Papoutsoglou EA et al (2020) Enabling reusability of plant phenomic datasets with MIAPPE 1.1. New Phytologist, 227, 260-273<br>(MIAPPE is a working group, not a software. It published a list of requirements, mentioned in the above paper)
Data analysis	Data analysis was performed using the open source R software (version 4.1.3), with modules and versions specified in the "method" section (SpATS, lme4, bnlearn, stats, Bayenv 2.0, statmod). No specific software was developed for this study. For architectural variables, methods refer to a published paper Perez et al 2019, Plant Cell Environment. GCTA software is fully described in Yang et al 2011). To our knowledge, only one version is available

For manuscripts utilizing custom algorithms or software that are central to the research but not yet described in published literature, software must be made available to editors and reviewers. We strongly encourage code deposition in a community repository (e.g. GitHub). See the Nature Research guidelines for submitting code & software for further information.

Data

Policy information about availability of data

All manuscripts must include a data availability statement. This statement should provide the following information, where applicable:

- Accession codes, unique identifiers, or web links for publicly available datasets
- A list of figures that have associated raw data
- A description of any restrictions on data availability

Data availability. Phenotypic and genotypic data are available at:
https://doi.org/10.15454/KLD0GH. They are presented with necessary metadata and explanation of the dataset. Supplementary File 1 provides the description of all

variables collected in every experiment, connected with the data files. Trait and environmental variables are described using the FAIR principle by stating trait entity, trait characteristic, method and unit. All these terms are mapped onto public ontologies, presented in Supplementary file 1 (as a spreadsheed) and in the published dataset (as csv files attached to data files for each experiment. Each figure of the main text is accompanied by an excel sheet that persents raw data. The soil information used for modelling is at ttps://esdac.jrc.ec.europa.eu/content/european-soil-database-v20-vector-and-attribute-data

Field-specific reporting

Please select the one below that is the best fit for your research. If you are not sure, read the appropriate sections before making your selection.

- [x] Life sciences
- [ ] Behavioural & social sciences
- [ ] Ecological, evolutionary & environmental sciences

For a reference copy of the document with all sections, see nature.com/documents/nr-reporting-summary-flat.pdf

Life sciences study design

All studies must disclose on these points even when the disclosure is negative.

Sample size	The number of accession was determined as the number of varieties that (1) were leaders of the market at the considered generation (2) falled within the considered maturity group (3) was compatible with the capacity of our phenotyping platforms (1600 plants divided in three treatments and 64 genotypes yields 8 repetitions). The final number of tested variety was the maximum number resulted from an optimisation of these constraits. Supplementary tables 1 and 2 provide the number of genotypes in each experiment, data collected and treatments. The rationale for representativeness of experiments and studied genotypes are presented in the first paragraph of results
Data exclusions	One field experiment was excluded because of very low heritability, which reached a level considered as unacceptable in most experimental analysis
Replication	Experiments had from 3 to 16 reps per hybrid, depending on the capacity of the considered platform. They were themselves considered as repetition in multi-site experiments. No repetitions were excluded. Heritabilities were calculated on all reported variables
Randomization	Experimental designs all included randomization, most often with alpha lattice designs (stated for each experiment in the method section)
Blinding	Not applicable, the design of multiple experiments was incompatible with blinding because of its complexity, with the same genotypes in different fields and different platforms. Blinding would have led to a high probability of errors

Reporting for specific materials, systems and methods

We require information from authors about some types of materials, experimental systems and methods used in many studies. Here, indicate whether each material, system or method listed is relevant to your study. If you are not sure if a list item applies to your research, read the appropriate section before selecting a response.

Materials & experimental systems

n/a	Involved in the study	
x		Antibodies
x		Eukaryotic cell lines
x		Palaeontology and archaeology
x		Animals and other organisms
x		Human research participants
x		Clinical data
x		Dual use research of concern

Methods

n/a	Involved in the study	
x		ChIP-seq
x		Flow cytometry
x		MRI-based neuroimaging
